# Supplementary material for: Comparative multiomics analysis of cell physiological state after culture in a basket bioreactor
Source: Sci Rep. 2022 Nov 23;12:20161. doi: 10.1038/s41598-022-24687-4 (PMC9686226; doi:10.1038/s41598-022-24687-4)
Supplement: Supplementary file 1 — Supplementary Information 1. [file 41598_2022_24687_MOESM1_ESM.zip › raw data/Transcriptomic raw data/6.Enrichment/1.GO/TE1vsTC1/GSEA/TC1/LIPASE_ACTIVITY/LIPASE_ACTIVITY(GO_0016298).html]

Details for gene set LIPASE\_ACTIVITY(GO:0016298)[GSEA]


|  || Dataset | readcount.sample |
| Phenotype | sample.cls |
| Upregulated in class | TC1 |
| GeneSet | LIPASE\_ACTIVITY(GO:0016298) |
| Enrichment Score (ES) | -0.19092013 |
| Normalized Enrichment Score (NES) | -1.1454737 |
| Nominal p-value | 0.0 |
| FDR q-value | 0.7420958 |
| FWER p-Value | 1.0 |
Table: GSEA Results Summary

  

Fig 1: Enrichment plot: LIPASE\_ACTIVITY(GO:0016298)
  
Profile of the Running ES Score & Positions of GeneSet Members on the Rank Ordered List

  

| PROBE | DESCRIPTION   (from dataset) | GENE SYMBOL | GENE\_TITLE | RANK IN GENE LIST | RANK METRIC SCORE | RUNNING ES | CORE ENRICHMENT || 1 | 103217592 | PLCL1 |  |  | 491 | Infinity | -0.0213 | Yes |
| 2 | 103246591 | PLCB4 |  |  | 4289 | 1.487 | -0.0911 | Yes |
| 3 | 103246657 | PLCB1 |  |  | 4679 | 1.438 | -0.0120 | Yes |
| 4 | 103216291 | PLCE1 |  |  | 5504 | 1.349 | 0.0416 | Yes |
| 5 | 103241730 | PLCL2 |  |  | 9445 | 1.057 | -0.0635 | Yes |
| 6 | 103245987 | PLCG1 |  |  | 11145 | 0.947 | -0.0758 | Yes |
| 7 | 103246100 | PLA2G4B |  |  | 11866 | 0.902 | -0.0475 | Yes |
| 8 | 103236105 | PLA2G12A |  |  | 11951 | 0.897 | 0.0089 | Yes |
| 9 | 103243297 | PLCD3 |  |  | 12078 | 0.890 | 0.0630 | Yes |
| 10 | 103233299 | PLCB3 |  |  | 12991 | 0.833 | 0.0781 | Yes |
| 11 | 103234777 | LIPE |  |  | 13663 | 0.791 | 0.1012 | Yes |
| 12 | 103216845 | SMPD4 |  |  | 14120 | 0.762 | 0.1320 | Yes |
| 13 | 103233371 | PLCG2 |  |  | 14829 | 0.719 | 0.1486 | Yes |
| 14 | 103221069 | PLCD1 |  |  | 16179 | 0.630 | 0.1306 | No |
| 15 | 103241439 | PLCH1 |  |  | 17879 | 0.500 | 0.0882 | No |
| 16 | 103230418 | PLA2G4A |  |  | 18290 | 0.461 | 0.1008 | No |
| 17 | 103217873 | PLCD4 |  |  | 19159 | 0.376 | 0.0873 | No |
| 18 | 103225758 | PLCH2 |  |  | 19598 | 0.328 | 0.0897 | No |
| 19 | 103235528 | PLA2G4C |  |  | 19975 | 0.276 | 0.0915 | No |
| 20 | 103245842 | PLCB2 |  |  | 20144 | 0.251 | 0.1008 | No |
| 21 | 103245774 | PLA2G4F |  |  | 21401 | 0.000 | 0.0447 | No |
| 22 | 103245777 | PLA2G4E |  |  | 22010 | 0.000 | 0.0175 | No |
Table: GSEA details
[plain text format]

  

Fig 2: LIPASE\_ACTIVITY(GO:0016298)
  
Blue-Pink O' Gram in the Space of the Analyzed GeneSet

  

Fig 3: LIPASE\_ACTIVITY(GO:0016298): Random ES distribution
  
Gene set null distribution of ES for
 **LIPASE\_ACTIVITY(GO:0016298)**
